# Management of an infection from NDM-1-producing *Escherichia coli* with susceptibility to aztreonam

**DOI:** 10.1128/asmcr.00173-25

**Published:** 2025-11-17

**Authors:** Alison M. Hixon, Sumanth Gandra, Rebekah E. Dumm

**Affiliations:** 1 Division of Infectious Disease, Washington University, St. Louis, Missouri, USA; 2 Pathology & Immunology, Washington University School of Medicine 12275 , St. Louis, Missouri, USA; Vanderbilt University Medical Center, Nashville, Tennessee, USA

**Keywords:** antibiotic resistance, NDM, aztreonam

## Abstract

**Background:**

Infections with New Delhi metallo-beta-lactamase (NDM)-producing Enterobacterales are increasingly reported in the United States, with some isolates being reported susceptible to aztreonam (ATM) alone. However, data for the use of ATM as monotherapy are lacking, and current guidelines recommend the use of cefiderocol (FDC) or ceftazidime-avibactam (CZA) plus ATM for severe infections due to concern for co-production of serine beta-lactamases.

**Case Summary:**

Here, we report a case of necrotizing soft tissue and surgical hardware infection in an immunocompromised patient with NDM-1-producing *Escherichia coli* that was susceptible to ATM, who was successfully treated with ATM + CZA followed by transition to ATM and minocycline.

**Conclusion:**

This case demonstrates the potential efficacy of ATM therapy, at least for part of the treatment course. Further studies are needed regarding treatment outcomes for ATM-susceptible NDM-producing Enterobacterales infections.

## INTRODUCTION

New Delhi metallo-beta-lactamase (NDM), a carbapenemase first identified in 2008, has emerged as a significant public health threat due to its ability to broadly hydrolyze beta-lactam antibiotics and its increasing global prevalence (1). Monobactams, such as aztreonam (ATM), retain activity against NDM-producing organisms; however, co-occurring serine beta-lactamases hydrolyze ATM and render it inactive against the organism (1, 2). Treatment options for NDM-containing Enterobacterales are therefore often very limited. Successful treatment has been reported with cefiderocol (Fetroja; FDC) alone or ATM combined with avibactam (AVI) through administration of ceftazidime-avibactam (CZA) to overcome serine beta-lactamases if present (3–6). However, these agents are expensive, require prolonged infusion durations with multiple times a day, making them not suitable for outpatient parental therapy at home or at skilled nursing facilities due to staffing issues and therefore require prolonged hospital stays. Recently, ATM-AVI (Emblaveo) received FDA approval, which has activity against NDM-producing organisms but with similar challenges regarding availability and administration of the antibiotic (7).

There has been a recent surge of infections with NDM-possessing Enterobacterales in the US, and, in line with this, we have been seeing more cases involving NDM in our hospital system (8, 9). Here we report a case of necrotizing soft tissue and surgical hardware infection in an immunocompromised patient with *Escherichia coli* possessing NDM-1, where the organism tested as susceptible to ATM alone. In such cases, there is limited guidance in the literature regarding ATM monotherapy or in combination with non-beta-lactam antibiotics. This patient initially received ATM + CZA, followed by transition to ATM and minocycline (MIN), with successful treatment of her infection. Here we discuss the report and local prevalence of ATM-susceptible NDM-producing Enterobacterales isolates and explore the complexities in navigating antibiotic selection in these cases.

## CASE PRESENTATION

This is the case of a 64-year-old woman with recurrent cervical cancer with metastases to the lungs, right humerus, and scalp on treatment with carboplatin, paclitaxel, bevacizumab, and pembrolizumab. Her other medical history included hypertension and chronic kidney disease. Social history was notable for living in the state of Missouri with her husband. She had no pets or animal exposures. She had no travel in the year preceding her presentation and no close contact with anyone traveling to regions of the world where NDM-producing organisms are considered endemic (10). She had no history of any form of substance use.

**Fig 1:**
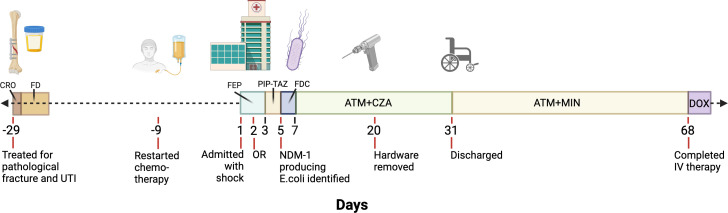
Timeline of patient history and antibiotic treatment. The timeline starts at day 1 with the patient’s admission to the hospital with septic shock. Twenty-nine days prior to the admission for septic shock, she suffered a pathological fracture of the right humerus requiring stabilization with hardware placement and was also treated for a suspected urinary tract infection (UTI) with ceftriaxone (CRO) and nitrofurantoin (FD). Nine days prior to the admission for septic shock, she restarted chemotherapy. On admission for septic shock with concern for necrotizing skin infection at her right arm surgical site, antibiotic coverage against gram-negative bacilli (GNB) consisted of cefepime (FEP). She was taken to the operating room (OR) on hospital day 2. On hospital day 3, GNB coverage was transitioned to piperacillin-tazobactam (PIP-TAZ). When cultures from right arm debridement identified an NDM-producing Enterobacterales, she was switched to FDC on hospital day 5 (day 3 of culture). After failure to clinically improve on FDC, she was transitioned to ATM plus CZA. She was also on IV MIN from hospital day 7 to 15 while awaiting susceptibility confirmation for ATM + CZA (not shown). She underwent full right arm hardware removal on admission day 20. She was discharged after 31 days inpatient on ATM and oral MIN. She completed IV therapy 68 days after admission and was transitioned to suppressive doxycycline (DOX). Antibiotics targeted against Gram-positive and anaerobic organisms are not shown. Created in BioRender. https://BioRender.com/skegqd8.

Shortly after starting chemotherapy, she suffered an acute pathological fracture of the right humerus at the site of her known metastasis (Fig. 1). She underwent open reduction and internal fixation of the pathological fracture, requiring extensive hardware placement given the abnormal bony architecture in the context of her malignancy. During that admission, she was found to have a urine culture positive for pan-susceptible *E. coli* that was treated with one dose of ceftriaxone (CRO) followed by 4 days of nitrofurantoin (FD). On post-operative follow-up, her surgical site appeared to be healing well without any complications. Given her reassuring examination, chemotherapy was restarted.

Nine days after receiving chemotherapy and approximately one month since her pathological fracture, she developed fever, chills, nausea, and vomiting, so she was admitted to the hospital and found to be in septic shock (Fig. 1). On exam, her right arm surgical site was dusky in appearance with dehiscence of skin at the incision line with tenderness to palpation concerning for a necrotizing soft tissue infection. She was leukopenic at 0.9 K/mm^3^ with an absolute neutrophil count of 0.4 K/mm^3^. ESR was 96 mm/hr with CRP 430.4 mg/L. She was initially treated with cefepime (FEP) 1,000 mg every 24 h (Q24H), linezolid 600 mg Q12H, and metronidazole 500 mg Q8H. She was taken for operative debridement of the right arm on hospital day 2. The operative report described necrotic skin, muscle, and bone requiring excision down to the level of the implanted hardware. Operative cultures initially grew methicillin-resistant *Staphylococcus aureus* (MRSA), vancomycin-sensitive *Enterococcus faecalis* (VSE), and *Klebsiella oxytoca*. The ICU team transitioned antibiotics to piperacillin-tazobactam (PIP-TAZ) 2.25G Q12H and linezolid on hospital day 3. On hospital day 5, she continued to require pressors. That same day, which was day 3 of the operative culture, an *E. coli* was reported that tested positive by the modified Carbapenem Inactivation Method (mCIM) and with *bla_NDM-1_* detected by CarbaR (Cepheid, Sunnyvale, CA). Notably, the CarbaR was negative for other serine carbapenemases (ie *bla_OXA48_* and *bla_KPC_*). Infectious Disease (ID) was consulted and recommended stopping PIP-TAZ and empirically starting FDC 1,000 mg Q8H to cover the *E. coli* plus metronidazole 500 mg Q12H. Linezolid was changed to daptomycin 950 mg Q24H for the MRSA and VSE.

Despite the change to FDC, the patient developed increasing pressor requirements, and the *E. coli* was sent for FDC susceptibility testing at a referral laboratory. The *E. coli* was susceptible to ATM, MIN, and doxycycline by a combination of in-house disk and gradient diffusion susceptibility testing methods. Given the patient’s clinical status and unknown result of FDC testing, the ID team recommended switching from FDC to empiric ATM 2,000 mg Q6H plus CZA 2.5G Q8H on hospital day 7. Formal susceptibility testing was also requested for ATM + CZA from the referral laboratory. MIN 200 mg Q12H IV was also added given the clinical acuity and while awaiting the confirmatory testing.

Within 24 h of starting ATM + CZA and MIN, pressor requirements began to improve. Metronidazole was stopped on hospital day 11. On hospital day 12, the referral lab MIC testing for *E. coli* was reported as susceptible to ATM (MIC two mcg/mL) and ATM + CZA (MIC one mcg/mL; MIC >64 mcg/mL for CAZ-AVI alone) and resistant to FDC (MIC 32 mcg/mL) by broth microdilution. The MICs for ATM and ATM + CZA were similar, indicating no additional advantage of ATM + CZA over ATM alone. A summary of the microbiological susceptibility profile can be found in Table 1. Clinical and Laboratory Standards Institute (CLSI) standards of susceptibilities were used throughout this report unless otherwise noted.

**TABLE 1:** Antibiotic susceptibilities of the NDM-1-producing *E. coli^b^*

Antibiotic	Interpretation^*a*^	MIC (µg/mL)
Amikacin	Resistant	
Ampicillin	Resistant	
Ampicillin with sulbactam	Resistant	
ATM	Susceptible	2
ATM with CZA	Susceptible	1
Cefazolin	Resistant	
FEP	Resistant	
Cefiderocol	Resistant	32
Ceftazidime	Resistant	
CZA	Resistant	>64
CRO	Resistant	
Ciprofloxacin	Resistant	
Doxycycline	Susceptible	
Eravacycline	Susceptible	
Ertapenem	Resistant	
Gentamicin	Resistant	
Imipenem	Resistant	
Imipenem-relebactam	Resistant	
Levofloxacin	Resistant	
Meropenem	Resistant	
Meropenem-vaborbactam	Intermediate	
MIN	Susceptible	
PIP-TAZ	Resistant	
Tobramycin	Resistant	
Trimethoprim with sulfamethoxazole	Resistant	

^*a*^ Interpretations of susceptibility were based on CLSI standards.

^*b*^ MIC – minimum inhibitory concentration; mL – milliliter; µg – microgram.

Through shared decision making with the medical teams, the patient and her family elected to remove the hardware with hopes for limb salvage with the goal to eventually continue chemotherapy. MIN was stopped on hospital day 15. After stabilization, the patient underwent complete hardware removal on hospital day 20. Tissue cultures from this operation grew MRSA only. On hospital day 25, the patient underwent hardware re-implantation and flap coverage. She was continued on ATM + CZA and daptomycin until hospital discharge. Given the difficulties in continuing CZA outpatient due to the cost and the duration of infusions (3 h per dose), as well as the culture indicating susceptibility to ATM alone, she was discharged to a long-term acute care facility on day 31 on ATM, oral MIN, and daptomycin with recommendations to continue this treatment for 6 weeks from source control. At the time of discharge, she was afebrile and hemodynamically stable. Her neutropenia had resolved, and her leukocyte count was 4.3 K/mm^3^. Inflammatory markers were still elevated; however, they improved when compared to admission, with ESR mm/hr and CRP being 92 and 231.2 mg/L, respectively.

She was seen in the outpatient ID clinic 3 weeks after discharge and was doing well. After completion of IV therapy, she was transitioned to suppression with doxycycline to cover the NDM-1 *E. coli* and MRSA and amoxicillin-clavulanate to cover the VSE and *K. oxytoca* in the prior cultures while pursuing further chemotherapy given concern for hardware replacement in abnormal bone that may not have received sufficient treatment before reimplantation.

Two months later, she developed pneumonia due to influenza A and non-COVID coronavirus requiring intubation. Despite aggressive treatment, her respiratory function did not improve. She was transitioned to comfort measures and passed away. Permission to share her case was obtained posthumously from her family.

## DISCUSSION

Treatment of NDM-producing Enterobacterales represents a significant challenge due to their broad hydrolytic activity against beta-lactam antibiotics. Guidance on the treatment of multi-drug-resistant infections from the Infectious Disease Society of America (IDSA) and the European Society of Clinical Microbiology and Infectious Disease (ESCMID) recommend ATM + CZA or FDC as preferred agents for severe infections when NDMs are present (11, 12).

To briefly summarize the data for these recommendations, several *in vitro* studies of Enterobacterales possessing NDM and other MBLs have demonstrated susceptibility to extended-spectrum beta-lactam and beta-lactam/beta-lactamase antibiotics (6, 13–17). Case studies and series have also demonstrated successful treatment of infections with MBL-producing Enterobacterales with a combination of ATM and CZA (4, 5). One study analyzed the outcomes of 102 patients with MBL infections (NDM included with other types of MBLs outside of the scope of this article) (4). Those treated with ATM + CZA had significantly lower rates of clinical failure and mortality compared to treatment with other susceptible antibiotics, which included regimens with colistin, aminoglycosides, tigecycline, and ATM typically in combination with at least one other agent. Given the promising results of field tests with ATM + CZA, guidance has been developed on timing of administration of both medications with close monitoring given concerns for potential toxicity given the combination of a monobactam and beta-lactam (12). Finally, FDC was approved by the FDA in 2019 (18). The APEKS-NP (NCT03032380) and CREDIBLE-CR (NCT02714595) demonstrated efficacy of FDC for non-carbapenemase producing multidrug resistant organisms and carbapenemase producing organisms including MBLs, respectively (19, 20).

There are several limitations, however, to ATM + CZA and FDC. These agents require prolonged infusion durations (3 h) multiple times per day (typically every 8 h for normal renal function), can be extremely cost-prohibitive, and are often difficult to manage as outpatient parenteral antibiotic therapy (OPAT) if prolonged treatment durations are required. In our experience, even discharge on these antibiotics to nursing facilities is often impossible given the cost and staffing requirements to provide such time-intensive therapy.

Furthermore, not every NDM-producing Enterobacterales appears to require the use of AVI, at least *in vitro*. A brief review of recent publications describing susceptibilities of carbapenemase-producing Enterobacterales, including MBL and NDMs to ATM report susceptibility (MIC ≤4 mcg/mL) (21) rates between 0-22%, with an average across these publications of 18% (Table 2) (3, 4, 6, 13, 14, 22, 23). Of note, most of these publications used CLSI standards except for one study using European Committee on Antimicrobial Susceptibility Testing (EUCAST) standards. In our hospital system, the number of NDM-producing isolates has doubled in recent years, with a total of 33 NDM-producing Enterobacterales isolated from January 2021 to August 2025. Of these, 27% have tested susceptible to ATM by disk diffusion, which is notably higher than the published average.

**TABLE 2:** Prevalence of ATM susceptibility in Carbapenemase and metallobeta-lactamase-producing enterobacterales^*c*^

Publication	Total (N)	S - ATM (N)	S - ATM (%)	Type of resistance
Sader et al. (23)^*a*^	120	3	2.5	CRE-producing Enterobacterales, including MBL and OXA-48-like producing organisms.
Emeraud et al. (3)^*a*^	58	0	0	MBL-producing Enterobacterales, including NDM, VIM, IMP.
Falcone et al. (4)^*b*^	102	7	7.3	MBL-producing Enterobacterales including NDM, VIM, IMP.
Bhatnagar et al. (13)^*a*^	64	4	6.25	MBL-producing Enterobacterales including NDM, VIM, IMP.
Rossolini et al. (1,4)^*a*^	1,607	354	22	CRE-producing Enterobacterales, including MBL, OXA, KPC producing organisms.
Harris et al. (22)^*a*^	322	54	16.8	NDM-producing Enterobacterales.
Tao et al. (6)^*a*^	61	6	9.8	MBL-producing Enterobacterales, including NDM, VIM, IMP.
BJH data, unpublished^*a*^	33	9	27	NDM-producing Enterobacterales
**Total**	2,367	437	18.5	

^*a*^ CLSI standards used for susceptibility interpretation.

^*b*^ EUCAST standards used for susceptibility interpretation.

^*c*^ ATM – aztreonam; CRE – carbapenemase; IMP – imipenemase; MBL – metallo-beta-lactamase; NDM – New Delhi beta-lactamase; S – susceptible; VIM – Verona-integron encoded metallo-betalactamase.

These data raise the possibility that ATM without CZA or AVI could play a larger role in treatment when no other beta-lactamases are present, especially when there are contraindications or barriers to treatment with the current standard of care. The literature provides limited guidance on treatment outside the use of FDC or ATM + CZA. Or, as was demonstrated in our case study, ATM + CZA could be used during critical or acute illness followed by consolidation to ATM, with or without an additional non-beta-lactam antibiotic if available, such as MIN in this case. The optimal timing of such a transition is unclear, but could be considered after achieving certain metrics, such as clinical stabilization off pressors, after surgical debridement or source control, or when ready for discharge.

Regarding prior studies suggesting higher risk of treatment failure with antibiotic regimen other than ATM + CZA (4, 5, 24), the total number of these cases was small, and susceptibility to ATM and the presence of other beta-lactamases in those cases was not delineated. In Falcone et al., many of the other antibiotic regimens contained colistin, with only 7 cases being treated with ATM alone or in combination (aminoglycoside, fosfomycin) (4). Our case highlights that more research is needed to understand when and how ATM without AVI should be used against ATM-susceptible NDM-producing Enterobacterales infections.

Lastly, in February 2025, the US Food and Drug Administration (FDA) approved the fixed-dose combination of ATM-AVI based on the phase 2 REJUVENATE and phase 3 REVISIT trials (25, 26). Rates of MBL and NDM infection were not described in the REVISIT study but were described in the concurrent ASSEMBLE trial (NCT03580044). In ASSEMBLE, 15 patients with MBL infections receiving ATM-AVI (*N* = 12) had a 41.7% rate of cure versus a 0% rate of cure with best available therapy (*N* = 3), although susceptibility to ATM alone was not reported (27). While ATM-AVI is promising, it is likely to suffer from the same drawbacks as ATM + CZA and FDC, with currently limited availability, high cost, and long (3 h) infusion times.

We hope this case helps to expand the discussion on other treatment options for NDM possessing Enterobacterales that are susceptible to ATM. If current trends continue in our hospital system and the US, we anticipate seeing many more such cases in the years to come. The limitations of our study include that this is a case report in which the patient received nearly a month of treatment ATM + CZA prior to transition to ATM + MIN, and it is possible that the patient was already cured of the NDM infection at the time the ATM outpatient therapy was started. We did not perform whole genome sequencing to rule out the presence of other serine beta-lactamases (ESBLs and AmpC) in our isolate.
